# UnbiasedDTI: Mitigating Real-World Bias of Drug-Target Interaction Prediction by Using Deep Ensemble-Balanced Learning

**DOI:** 10.3390/molecules27092980

**Published:** 2022-05-06

**Authors:** Aida Tayebi, Niloofar Yousefi, Mehdi Yazdani-Jahromi, Elayaraja Kolanthai, Craig J. Neal, Sudipta Seal, Ozlem Ozmen Garibay

**Affiliations:** 1 Department of Industrial Engineering and Management Systems, University of Central Florida, Orlando, FL 32816, USA; aida.tayebi@knights.ucf.edu (A.T.); niloofar.yousefi@ucf.edu (N.Y.); yazdani@knights.ucf.edu (M.Y.-J.); 2 Advanced Materials Processing and Analysis Center, Department of Materials Science and Engineering, University of Central Florida, Orlando, FL 32816, USA; elayaraja.kolanthai@ucf.edu (E.K.); craig.neal@ucf.edu (C.J.N.); sudipta.seal@ucf.edu (S.S.); 3 College of Medicine, Bionix Cluster, University of Central Florida, Orlando, FL 32816, USA

**Keywords:** drug-target interaction, ensemble learning, deep learning, machine learning, spike protein, SARS-CoV-2, ACE2 receptor

## Abstract

Drug-target interaction (DTI) prediction through in vitro methods is expensive and time-consuming. On the other hand, computational methods can save time and money while enhancing drug discovery efficiency. Most of the computational methods frame DTI prediction as a binary classification task. One important challenge is that the number of negative interactions in all DTI-related datasets is far greater than the number of positive interactions, leading to the class imbalance problem. As a result, a classifier is trained biased towards the majority class (negative class), whereas the minority class (interacting pairs) is of interest. This class imbalance problem is not widely taken into account in DTI prediction studies, and the few previous studies considering balancing in DTI do not focus on the imbalance issue itself. Additionally, they do not benefit from deep learning models and experimental validation. In this study, we propose a computational framework along with experimental validations to predict drug-target interaction using an ensemble of deep learning models to address the class imbalance problem in the DTI domain. The objective of this paper is to mitigate the bias in the prediction of DTI by focusing on the impact of balancing and maintaining other involved parameters at a constant value. Our analysis shows that the proposed model outperforms unbalanced models with the same architecture trained on the BindingDB both computationally and experimentally. These findings demonstrate the significance of balancing, which reduces the bias towards the negative class and leads to better performance. It is important to note that leaning on computational results without experimentally validating them and by relying solely on AUROC and AUPRC metrics is not credible, particularly when the testing set remains unbalanced.

## 1. Introduction

Drug-target interaction (DTI) prediction through in vitro experiments can be costly and time-consuming. In silico approaches, on the other hand, can save time and cost while improving the efficiency of drug discovery. An advantage of in silico approaches is their practical applicability in drug repurposing applications, where previously known drugs can be screened for new treatments and purposes. This is according to the fact that repurposed drugs are already proven to be safe, in many cases are FDA-approved, and they have passed the clinical trials, where the side effects and other characteristics of the drugs have been studied [1]. Therefore, the computational screening of these drugs for a given target of interest can provide a list of candidates for further in vitro and in-lab experiments.

Two types of in silico methodologies have been proposed to analyze drug-target interactions. The first approaches treat the DTI challenge as a binary classification task, with the goal of predicting whether or not a drug-target pair interacts. The second approach frames it as a regression task, which predicts the binding affinity or strength of interaction between a drug and its target. These in silico approaches can vary from ligand-based (receptor-based) [2] methods to machine learning-based methods. Docking simulations, for example, are a successfully utilized method in receptor-based approaches, which are very expensive, require 3D protein structures, and are not always available [3]. Machine learning (ML)-based methods have generally outperformed traditional ligand-based models, since ligand-based techniques are based on the similarity between the candidate ligand for a given target protein, and the ligand information is not available for all target proteins. In machine learning methods, drugs and targets are represented by a set of descriptors and features, and the model learns from this encoded information. Different types of features, including molecular fingerprints and amino acid composition, have been proposed and utilized in different studies. However, it is a challenging task to find the most effective representation for drugs and proteins and translate them into numerical features, and yet avoid the high dimensionality issue [4]. Nevertheless, there are several publicly available resources, such as DrugBank [5], ChEMBL [6], and BindingDB [7], that provide information on compounds and their targets in order to create feature representations for both drugs and proteins.

An important piece of information here is the strength of the interaction in a given drug-target pair, which is measured by the binding affinity value. The binding affinity can be in the form of Kd (dissociation constant) [8,9], Ki (inhibition constant) [10,11], or IC50 (half-maximal inhibitory concentration) [12]. Most of the computational methods simplify the problem of DTI prediction by framing it as a binary classification task, where the goal is to determine whether or not a drug–target (DT) pair interacts. These methods, typically, set a threshold on the binding affinity values and divide the data into two classes: a positive class with low Kd, Ki, or IC50 values, which represents the interacting pair, and a negative class with higher Kd, Ki, or IC50 values, which includes the non-interactive pairs [13,14]. One major limitation of these classification approaches is that, in all DTI-related datasets, the number of negative instances are far higher than positive instances, leading to the class imbalance issue [15]. This, in turn, results in the creation of a classifier, which is biased towards the majority class (negative class), whereas the minority class (interacting pairs) is of interest in drug-repurposing applications. There are only a few studies that have addressed the class imbalance issue in drug-target interaction prediction tasks. Several balancing methods such as random undersampling (RUS) [15,16,17,18,19,20,21], synthetic oversampling (e.g., SMOTE [22]) [17,23,24], balanced random sampling (BRS) [21,25] and cluster-based undersampling (CUS) [18,20] have been employed in the DTI literature. Other studies rely solely on evaluation metrics such as the F1 score or the area under the receiver operating characteristic (ROC) curve to alleviate the bias in their model prediction. However, this can be misleading in many cases, as the bias towards the majority class still exists in the predictions.

## 2. Background

To address the imbalance issue, Ezzat et al. [15] first divided the problem into two sub-problems: between-class imbalance (the imbalance ratio between the negative and positive class) and within-class imbalance (the imbalance between well-represented and less-represented interactions) and proposed an ensemble of decision trees. They used oversampling of the less-represented interactions for within-class imbalance and random undersampling (without replacement) for between-class imbalance issues and trained different decision trees using this balanced data. Their results showed better performance compared to other traditional machine learning methods such as SVM and nearest neighbor. Ezzat et al. [16] extended their previously proposed method by solely focusing on between-class imbalance and trying different variants (same training set, bagging, bagging on negatives only and different negative sets) to aggregate the decision trees. They also showed the effect of the number of decision trees on the total AUC. Rayhan et al. [20] proposed the AdaBoost algorithm for DTI prediction using evolutionary and structural features. In this study, random undersampling and cluster undersampling methods were utilized to balance the datasets. Their results showed that, in terms of area under the ROC curve, random sampling is slightly better than cluster-based sampling in half of the datasets (enzymes and ion channel), but in the other half (GPCRs and nuclear receptors), it the opposite was true. Recently, Mahmud et al. [17] proposed a computational method for the DTI problem using the XGBoost classifier. The protein sequence was vectorized through feature extraction methods called PSSM-Bigram, DP-PseAAC and AM-PseAAC. The drug compounds were vectorized through molecular substructure fingerprint (MSF). Subsequently, balancing techniques including random sampling and SMOTE were utilized. Finally, the XGBoost algorithm was applied to predict DTIs. Another similar work was done by Mahmud et al. [18], where more features were added to the protein representations. Furthermore, following data balancing, a modified dimensionality reduction technique was proposed, and the boosting algorithm was employed. Redkar et al. [23] used dipeptide composition to encode the protein, and molecular descriptors were utilized for drugs. Different traditional machine learning algorithms such as decision tree, KNN, SVM, XgBoost etc. were deployed using wrapper feature selection and SMOTE.

Although the aforementioned studies have provided improvements by adding a balancing step in predicting DTIs, some drawbacks still exist: (i) most of these studies do not focus on the imbalance issue itself, (ii) none of the mentioned studies have used deep learning models, which outperform conventional machine learning models, (iii) most of these studies have considered unknown interactions as negative samples, which can lead to ineffective prediction, and (iv) none of the aforementioned studies have provided in-lab experimental validation to show the impact of balancing. To overcome these issues, we use a computational-experimental framework, where the performance of our proposed model is compared against unbalanced models. In this paper, we address the class imbalance problem by utilizing a random undersampling technique. In order to minimize the discarded information from the negative class (majority class), we propose an ensemble of deep learning models, where the positive samples are kept constant for all the base learners, and we only perform random undersampling on the negative set. Unlike other studies, we only consider known interactions and experimentally validated negative samples. We use protein sequence composition (PSC) descriptors to represent the target protein. PSC descriptors are one of the most frequently used encodings in the sequence-based approaches. Additionally, we use the SMILES (Simplified Molecular-Input Line-Entry System) of the compounds to generate a feature vector representing the drug. Furthermore, we use ErG (Extended reduced Graphs) and ESPF (Explainable Substructure Partition Fingerprint) fingerprints to benefit from different embeddings, and then utilize a neural network for each embedding to extract the most discriminative features. These feature vectors are then concatenated and fed into another fully connected network to build each learner of the proposed model. Finally, all these deep learning models are aggregated to build the proposed ensemble model.

Our analysis shows that the proposed model outperforms the unbalanced models both computationally and experimentally in all metrics. The experimentally validated pairs were new unseen interactions and were not contained in the training dataset. These results indicated the effectiveness and the importance of balancing steps in DTI prediction tasks, which is not widely taken into account in DTI prediction studies.

## 3. Materials and Methods

### 3.1. Data

In this study, we use BindingDB, a publicly available benchmark dataset [7]. By limiting the bioactivity type to IC50 values only, we were able to use a subset of the dataset that contained 1,369,057 experimentally validated drug-target pair interactions, with 492,970 of those being labeled as positive. This was split into a training set of size 1,163,698 (85% of data) with 419,001 positive instances, and a test set of size 200,225 (15% of data). A threshold of 100 nM (PIC50 ⩾ 7) was deployed similar to [26] to split the dataset into positive (interacting) and negative (non-interacting) classes. Table 1 summarizes the statistics of the BindingDB dataset. The details of IC50 values, length of SMILES characters, and length of protein sequences are shown in Table 2.

### 3.2. Ensemble Methods

The ensemble learning approach includes training multiple models, called base models (or weak learners), for the same problem, and then aggregating them to achieve an ensemble model which is less sensitive to data, more accurate, and more robust than each individual base model. The main advantage of using ensemble methods is that different contributing models do not all make the same errors on the test set [27], therefore combining the predictions from multiple models. This results in better predictions than any single best model, and combining several base models together creates a more powerful ensemble model [28]. The first step to set up an ensemble learning method is to select the base models. The models used in the ensemble methods (base learners) can vary in different elements, as follows.

#### 3.2.1. Different Training Data

Base learners can vary in training data. In this group, the learners are homogeneous in structure, and depending on how the learners are trained (independently and in parallel or sequentially and in adaptive way), the ensemble approach is called bagging (short for bootstrap aggregation) or boosting. A popular approach is to resample the training dataset (this could be resampling with or without replacement) to construct different datasets, so the base learners trained on different datasets vary in predictions [29].

#### 3.2.2. Different Models

Base learners can vary in terms of building the model itself. This category of ensemble models includes training heterogeneous learners on the same data, but with varying initial conditions. This might not improve the generalization of the model, since the errors and the predictions made by different learners might be highly correlated due to being trained on similar data. An alternative approach to make the errors of the learners independent from each other is to alter configurations such as number of layers or nodes or different hyperparameters, such as the learning rate [30].

#### 3.2.3. Different Combinations of the Outputs

The outcome of each base learner in the ensemble model can vary in such a way that each model has an impact on the final result. These methods can vary from simple or weighted averages to complex 2nd- or 3rd-level meta-models. To add one step of complexity, a stacking approach is used by training a new meta-model to learn how to best combine heterogeneous learners that were trained in parallel [31].

### 3.3. Methodology

In this study, an ensemble of deep neural networks is proposed to address the problem of class imbalance and to reduce the bias towards negative interactions. The main idea is to aggregate the predictions from multiple deep learning models trained with a balanced dataset to get the best and the most unbiased prediction possible. To avoid high computational cost, the number of learners in the ensemble model is often kept small. In this study, we trained 16 deep neural network models with similar configurations but trained on different datasets. Despite the fact that two separate encoders are employed for drugs, the models can be deemed homogeneous, as they are trained independently and in parallel, using identical network architectures. The predictions of these base models are aggregated by taking the arithmetic mean on the output predicted probability of each model:

(1) $$\mathrm{Ensemble}\;\mathrm{of}\;\mathrm{balanced}\;\mathrm{models}\;=\;\frac{1}{M}\sum_{1}^{M}\mathrm{Output}\;\mathrm{Probability}\;\mathrm{of}\;\mathrm{Each}\;\mathrm{Learner}$$

where *M* is the number of base learners and the output is the predicted probability of that instance belonging to the positive class.

The pseudocode of the proposed method is shown in Algorithm algo. As presented in the algorithm, random undersampling was utilized following the works of [15,16,17,18,19,20,21]. The positive set remained constant for each model, and bagging was performed on the negative set. The only criterion for us was forming negative and positive sets of the same sizes. Eventually, the performance of the ensemble model was compared to the unbalanced models.

**Algorithm 1** Pseudocode of ensemble of classifiers as well as undersampling the negative set  **Input:**  *P*= Positive set (minority class samples in the dataset *D*)  *N*= Negative set (majority class samples in the dataset *D*)  *M*=Number of base learners in ensemble model  **Output:**    Ensemble = trained ensemble.    **for**

j∈{ErG,ESPF}

**do**        **for** 
i∈1→8

**do**            Randomly sample 
$N_{i}\in N$
: 
$\left|N_{i}\right|=\left|P\right|$
            
${\mathrm{Classifier}}_{i,j}$
 = train classifier using *P* and 
$N_{i}$
        **end for**  **end for**  

$\mathrm{Ensemble}=\frac{1}{M}\sum_{1}^{M}\mathrm{Output}\mathrm{Probability}\mathrm{of}\mathrm{Each}\mathrm{Learner}$


**Figure 1:**
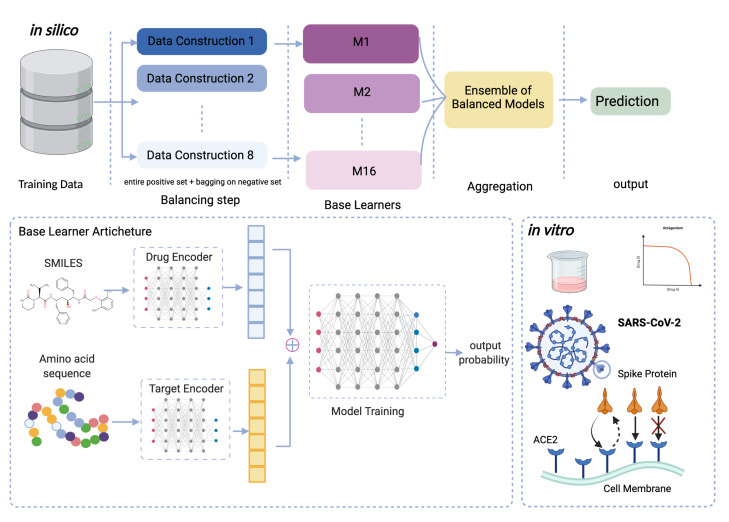
Framework of the proposed method to predict DTI. The in silico component starts with preprocessing and data construction. Each data construction includes the entire positive set and bagging on the negative set. These base learners are then aggregated and create the deep ensemble-balanced learning models. The output is the predicted probability of unknown interaction of a drug-target pair. The architecture of each individual learner is shown as a part of this framework, where the drug and protein representations are first extracted by utilizing neural networks from their corresponding SMILES and amino acid sequences, and then these encodings are concatenated and fed into the final neural network, where the model is trained. The in vitro component includes the validation part of our framework, where computational results of our proposed model are compared to the experimentally-measured DTI in the laboratory.

As shown in the schematic diagram in Figure 1, each individual learner consists of three deep learning modules: a module to encode the drug compound, a module to encode the protein target, and finally, one module to make predictions. Each encoder is a multilayer perceptron (MLP) with four linear layers with 1024, 256, 64, and 256 neurons. The drug encoder takes the SMILES sequence of the small molecules and returns a feature vector representing the corresponding drug. For drugs, two predefined fingerprints, namely ErG [32] and ESPF [33], are used separately to convert the SMILES into binary vectors. These embeddings then are fed into a fully connected network for feature extraction. Thus, depending on the model, either an ESPF vector with a size of 2586 or an ErG vector with a size of 315 enters the drug encoder, and a feature vector of 256 is kept. The target encoder takes the amino acid sequence of the protein as input and returns a feature vector corresponding to the target. Thus, the protein vector with a size of 8420 enters the protein encoder and is reduced to a feature vector of size 256. For targets, Protein Sequence Composition (PSC descriptors) [34] is used to convert the protein sequence into a binary vector. These embeddings are then fed into a fully connected network for feature extraction. Feature vectors for drug and protein including 256 features of ESPF/ErG along with 256 features of the protein (summing up to 512 features) are finally concatenated and fed into a fully connected network, which is an MLP with four linear layers with 1024, 1024, 512, and 1 neuron. This is a binary classifier, predicting whether or not a given drug-target pair interacts. The ReLu activation function is used in all layers. The details of the networks can be found in Table 3. All models are adapted from the DeepPurpose package [35].

### 3.4. Drug and Target Vectorization

#### 3.4.1. Protein Vectorization

Using Protein Sequence Composition (PSC) descriptors is a popular approach to represent a protein from its amino acid sequence. This embedding consists of three components: amino acid composition (AAC) [34], dipeptide composition (DC) and tripeptide composition (TC) [36]. AAC is the frequency of twenty amino acid residues in a protein sequence. Dipeptide composition is the frequency of a given dipeptide, which is a combination of two amino acids and consists of 400 values ( 20×20 ). Tripeptide composition is the frequency of three amino acid compositions, which consists of 8000 features ( 20×20×20 ). Thus, the feature vector of PSC has a total of 8420 descriptors (8000 + 400 + 20).

#### 3.4.2. Compound Vectorization

Simplified Molecular-Input Line-Entry System (SMILES) sequences of the compounds were used to represent the drugs. SMILES sequences contain information such as the presence of atoms, bonds, branching, aromaticity, ring structures, isotopes, etc. To featurize the SMILES, we tried four different fingerprints, including ErG, ESPF, Morgan and Daylight, and we decided to use the ErG and ESPF fingerprints, as they performed better than the other two hashing-based fingerprints, Morgan [37] and Daylight [38]. One of the drawbacks of these molecular descriptions is that it is difficult to map them back to the original sub-structure, which is due to the complex nature of hashing. Additionally, if there is an undesirable feature in the query structure, it is very likely to retrieve similar compounds with the same unwanted features.

*ErG:* Extended reduced graphs (ErG) [32] are a 2D fingerprint that act as an extension and modified version of the reduced graphs described by [39,40]. Gillet et al. [40] and Barker et al. [39] proposed the reduced graphs, which can be described as summaries of a substance’s major structural features. In reduced graphs, instead of using every single atom to generate descriptors, a more abstract description of chemical properties is used. Stief et al. [32] proposed a hybrid approach and combined these reduced graphs with binding property pairs [41]. In both reduced graphs and ErG, relevant features are captured; however, ErG generates a more general descriptor of the original features, and the focus is to capture the correct size and shape (encoding rings, substitution patterns, fused rings, and endcap groups). These modifications eventually result in a better descriptor in terms of the pharmacophoric properties, including the size, shape, and encoding of relevant molecular properties. As indicated in [32], ErG outperforms techniques such as Daylight fingerprints. Comparing to hashing-based fingerprints, ErG is capable of detecting more structurally diverse compounds [32].

*ESPF:* Recently, an interpretable fingerprint was proposed by Huang et al. [33], entitled Explainable Substructure Partition Fingerprint (ESPF), which was inspired by some studies in the domain of natural language processing such as [42]. Huang et al. [33] claims that there is a lack of explainability and interpretability in the existing fingerprints, such as hashing-based fingerprints, molecule-level fingerprints, expert-curated fingerprints such as PubChem [43], and MACCS [44]. Based on this study, these fingerprints do not effectively link the relevant sub-structures to specific features and do not indicate which functional group of drugs can map to some specific molecular properties. ESPF was proposed to tackle these issues and to make a tractable fingerprint that maps specific sub-structures to the prediction outcome. In ESPF, a predefined database of sub-word units is used that contains frequently recurring sub-structures (frequencies greater than 1500) of the CHEMBL dataset [6]. This is customized to result in an efficient predictive output. Given this database, ESPF breaks any unseen input SMILES into discrete pieces of sub-structures and then replaces them with the most credible subword units which were predefined in the mentioned database at hand. To be more specific, the original input is first replaced with the predefined tokens, which in turn, is translated into a fixed-sized binary vector, indicating the presence of sub-structures. This fingerprint has shown competitive performance against state-of-the-art fingerprints such as ECFP (Extended Connectivity Fingerprints) [37].

A summary of the used embeddings for both the protein and the compound can be found in Table 4.

### 3.5. Lab Validation

In this section, a set of candidate molecules are used to evaluate the performance of the computational models. Severe Acute Respiratory Syndrome Coronavirus 2 (SARS-CoV-2) started the COVID-19 pandemic in 2019. Ever since, the research community has been conducting research and clinical trials to discover drugs that bind to SARS-CoV-2. The SARS-CoV-2 spike protein interacts with host ACE2 receptors and enters the host cell. Thus, the inhibition of tripeptide formation plays a significantly important role in preventing the entry of the SARS-CoV-2 virus, and it is important to predict compounds that are able to bind to this complex. For this purpose, seven molecules were chosen, and an ELISA-type competition assay was performed. These molecules were tested using the ACE-2: SARS-CoV-2 Spike Inhibitor Screening Assay Kit. The ACE2 receptor was bound to a test plate where spike proteins were added to bind to receptor sites. This experiment was designed in a way that tests whether the compound inhibits and prevents the formation of ACE2 and spike complex or not and does not test which protein the inhibitor binds to. Therefore, the model predictions are reported for ACE2 and the spike protein separately. The results for the ACE2 receptor are reported, and the results for the spike protein can be found in Appendix A. The IC50 values of N-Acetyllactosamine, N-acetyl-neuraminic acid, 3 α ,6 α -Mannopentaose, N-glycolylneuraminic acid, 2-Keto-3-deoxyoctonate ammonium salt, cytidine-5-monophospho-N-acetylneuraminic acid sodium salt, and darunavi were calculated. All the compounds except 3 α ,6 α -Mannopentaose have IC50 values of under 30 nM. Based on our threshold, which is 100 nM, these are considered positive interactions. Details of the experiments can be found in Appendix A.

## 4. Results and Discussion

### 4.1. Evaluation Metrics

The area under the receiver operating characteristic curve (AUROC) and the area under the precision recall curve (AUPRC) are the two most widely used metrics in this domain. AUROC and AUPRC have become standard metrics to measure the performance of prediction models on unbalanced datasets, since these plots are independent of the chosen threshold. The ROC curve plots sensitivity against the false positive rate (1-Specifity) at various threshold values. Similarly, the PRC curve plots precision against recall (sensitivity) at various threshold values. A random classifier has an AUROC value of 0.5, and higher values of AUROC indicate better performance. Compared to AUROC, AUPRC is more sensitive to false positives [20], which makes it a more appropriate metric when there is a large skew in class distribution and in unbalanced datasets. Similar to AUROC, higher values of AUPRC correspond to better performance.

Sensitivity or recall (also known as the true positive rate) is the ratio of true positive samples to the total number of positive samples and is defined as follows:

(2) $$\begin{array}{c} \mathrm{Sensitivity}\;\left(\mathrm{Recall}\right)=\frac{TP}{TP+FN} \end{array}$$

Specificity or the true negative rate is the ratio of true negative samples to the total number of negative samples and is defined as follows:

(3) $$\begin{array}{c} \mathrm{Specificity}=\frac{TN}{TN+FP} \end{array}$$

Precision or PPV (positive predictive value) shows the percentage of accurate positive predictions for all positive predictions and is defined as follows:

(4) $$\begin{array}{c} \mathrm{Precision}=\frac{TP}{TP+FP} \end{array}$$

The F1-score is another important metric in the literature and is defined as follows:

(5) $$\begin{array}{c} F1-\mathrm{Score}=\frac{2*\mathrm{Precision}*\mathrm{Recall}}{\mathrm{Precision}+\mathrm{Recall}} \end{array}$$

### 4.2. Comparison of Results between Unbalanced Models vs. Proposed Method

The effectiveness of the balancing step through our proposed ensemble method is explained in this section. To compare the computational results of all models, we report the F1-score, AUPRC, and AUROC, as they are the most frequently reported metrics in DTI. As mentioned before, these metrics can be inadequate, and therefore, we also report recall (true positive rate), which is a useful metric to investigate the validity of our research hypothesis. Table 5 illustrates the performance of two unbalanced models versus our proposed model (ensemble of balanced models). The two unbalanced models were trained with the same set of features for protein (PSC) and two distinct set of features for drugs (ErG and ESPF) on the BindingDB dataset. The testing set consisting of 200,225 drug target pairs, of which only 72,074 are positive interactions (the class of interest), was kept constant for all three models. Using this dataset, ESPF-PSC achieved an AUROC value of 0.924, an AUPRC value of 0.879, an F1-score of 0.809, and a true positive rate of 0.797. Similarly, ErG-PSC obtained an AUROC value of 0.926, an AUPRC value of 0.876, an F1-score of 0.796 and a true positive rate of 0.809. In terms of the AUPRC and F1-score, the ESPF feature group seems to work slightly better than the ErG feature group. Additionally, in terms of the AUROC and true positive rate, the results are in favor of the ErG feature group. Our proposed model yielded an AUROC value of 0.952, an AUPRC value of 0.920, an F1-score of 0.838 and a true positive rate of 0.903. We can argue that the performance of our proposed model is consistent and our model yields significantly better results compared to the other two unbalanced models. Additionally, it outperforms the unbalanced models in all the metrics computationally.

**Figure 2:**
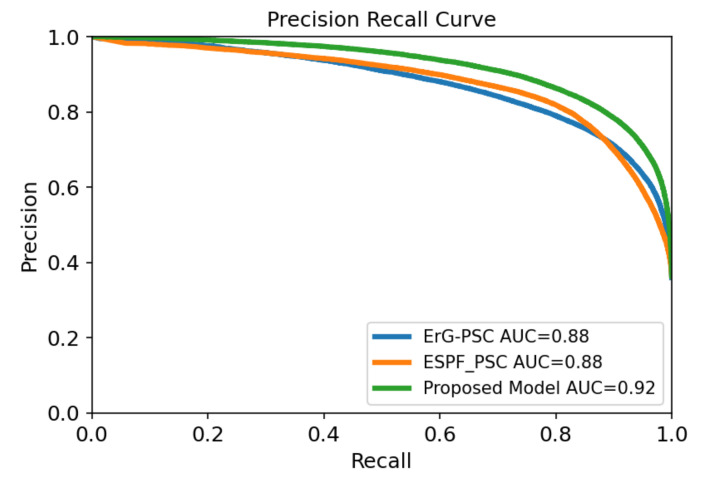
Precision recall curve yielded for two unbalanced models and our proposed method on the BindingDB dataset.

**Figure 3:**
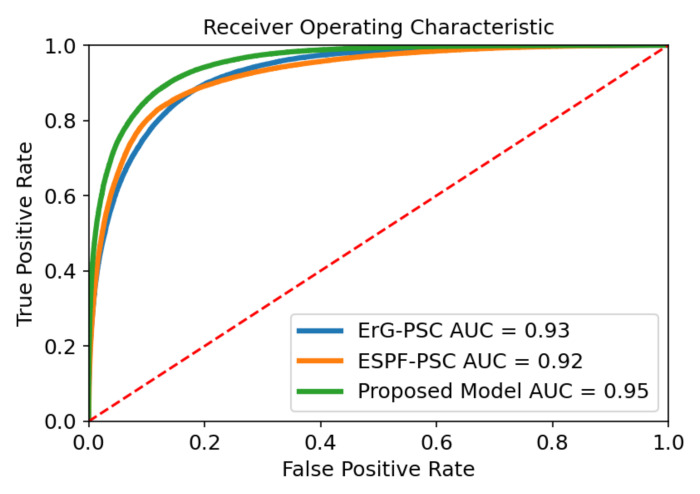
Receiver operating characteristic curve yielded for two unbalanced models and our proposed method on the BindingDB dataset.

We validated our analysis using lab experiments as well. Table 6 summarizes the lab experiments and prediction of the computational unbalanced and proposed models. As mentioned earlier, seven compounds were tested in the lab. The results of the lab experimental validation showed that one compound (3 α ,6 α -Mannopentaose) was inactive and the other 6 compounds were active against the complex of the spike protein and ACE2. We must mention that the tested pairs were new drug-target interactions and were not contained in the dataset on which the models were trained. As indicated in Table 6, the ESPF-PSC unbalanced model is completely biased and predicts negatives for all seven compounds. The ErG-PSC unbalanced model works slightly better, with 3 correct predictions, which indicates that ErG extracts better features compared to ESPF encoding. An interesting observation is that despite close AUPRC, AUROC, and F1 scores for the unbalanced models, their predictions deviated greatly from each other, which shows how these metrics can be often deceptive. Our proposed model was able to predict new drug-target interactions with a true positive rate of 0.833 and yielded 6 out of 7 correct predictions (5 true positives and 1 true negative); furthermore, the experimental results are consistent with computational results. It’s important to note that the proposed model is a combination of the two drug feature groups (ErG and ESPF) with the same target feature group (PSC) trained with balanced datasets, which means that the architecture used in neural networks in unbalanced models is identical to the architecture of each learner in the proposed ensemble learning, and the differences in results are solely the impact of data balancing and ensemble learning. The respective receiver operating characteristic and precision recall curves for three models are displayed in Figure 2 and Figure 3.

## 5. Conclusions

In this article, a computational framework is proposed along with experimental validations to predict drug-target interaction using a deep ensemble-balanced learning. The objective is to address the class imbalance problem in the DTI domain, which often leads to biased predictions. In contrast with the few previous studies considering balancing in DTI, this work focuses on the impact of balancing while maintaining other involving parameters constant. Additionally, lab experimental validations are provided. Based on the computational and experimental results, balancing is a critical step often neglected in DTI literature, which reduces the bias towards negative class and leads to better performance. It is important to note that relying on computational results without validating them through lab experiments is not adequate and cannot be credible. AUROC and AUPRC metrics, which are standard metrics in this domain, are not solely reliable, and other metrics, such as the true positive rate, should be considered, especially when the testing set is also unbalanced and the negative class is still the majority class. Our analysis shows that the proposed model achieves the highest performance compared to the same architecture trained on the BindingDB unbalanced dataset, both computationally and experimentally. Our findings will enrich the future research and suggest the studies in this domain to focus on this issue to train more realistic, reliable models that are compatible with real world data. In future work, we shall consider developing more advanced balancing techniques.

## Figures and Tables

**Table 1:** Dataset description and statistical information.

	Unique Drugs	Unique Targets	Total Pairs	Positive Pairs	Negative Pairs	Imbalance Ratio
BindingDB Dataset	679,118	5941	1,369,057	492,970	876,087	1.78

**Table 2:** Detailed IC50 values, lengths of SMILES characters and lengths of protein sequences.

	Bioactivity Type	IC50 Value			SMILES Sequence Length			FASTA Sequence Length		
		Max	Min	Avg	Max	Min	Avg	Max	Min	Avg
BindingDB Dataset	IC50	1 × 10^7^	0	3.79 × 10^4^	1.94 × 10^3^	2.0 × 10^0^	5.85 × 10^1^	7.18 × 10^3^	9.0 × 10^0^	7.07 × 10^2^

**Table 3:** Details of the neural networks.

Layer	ErG	ESPF	PSC	Final FC Network
First	Linear(315, 1024)	Linear(2586, 1024)	Linear(8420, 1024)	Linear(512, 1024)
2nd	Linear(1024, 256)	Linear(1024, 256)	Linear(1024, 256)	Linear(1024, 1024)
3rd	Linear(256, 64)	Linear(256, 64)	Linear(256, 64)	Linear(1024, 512)
4th	Linear(64, 256)	Linear(64, 256)	Linear(64, 256)	Linear(512, 1)

**Table 4:** Summary of structural features used for protein and fingerprint features for drugs.

Name	Description	Size	Feature Group
PSC	Amino acid composition up to 3-mers	8420	Target
ErG	2D pharmacophore descriptions for scaffold hopping	315	Drug
ESPF	Explainable Substructure Partition Fingerprint	2586	Drug

**Table 5:** Comparison of the computational results yielded for two unbalanced models and our proposed method on the BindingDB dataset.

	AUROC	AUPRC	F1-Score	Recall (TPR)
**Unbalanced Model 1 (ESPF-PSC)**	0.924	0.879	0.809	0.797
**Unbalanced Model 2 (ErG-PSC)**	0.926	0.876	0.796	0.809
**Proposed Model**	0.952	0.920	0.838	0.903

**Table 6:** Comparison of the two unbalanced models and our proposed method on in-lab experimental data.

Compound	Lab Results	Unbalanced Model 1	Unbalanced Model 2	Proposed Model
darunavir	P	N	N	P
2-keto-3-deoxynononic	P	N	N	N
Cytidine-5monophospho-N-acetylneuraminic	P	N	P	P
N-Glycolylneuraminic	P	N	P	P
N-acetyl-neuraminic	P	N	P	P
N-Acetyllactosamine	P	N	N	P
3 α ,6 α -Mannopentaose	N	N	N	N
Recall(TPR)		0	0.5	0.833

**Figure A1:**
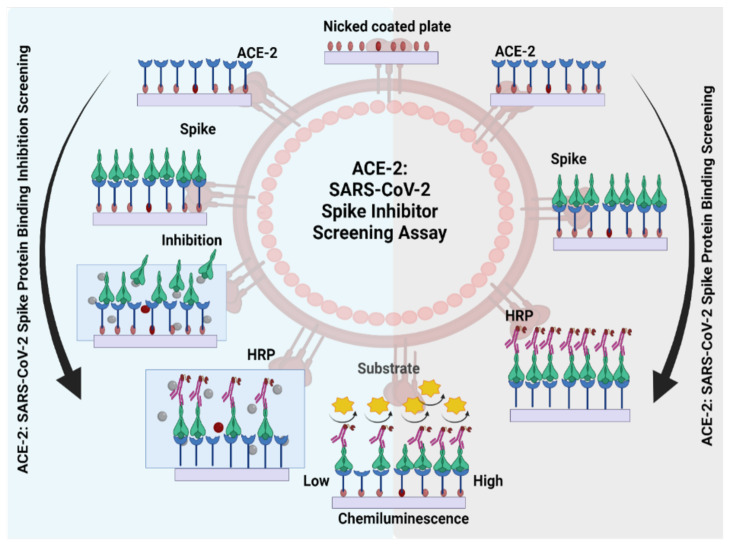
The schematic diagram indicates the binding between spike protein of the SARS-CoV2 and ACE2 protein on the cell membrane and how the inhibitor compounds work against this inhibition. ACE-2:SARS-CoV-2 spike protein binding screening (negative control): first, the immobilized ACE2 protein in a nickel-coated 96-well plate followed by enough spike protein added on top. In addition, the HRP-modified antibody is bound to the top of the protein, and then the catalyst substrates produce chemiluminescence. Seven molecules including N-Acetyllactosamine, N-acetyl-neuraminic acid, 3 α ,6 α -Mannopentaose, N-glycolylneuraminic acid, 2-Keto-3-deoxyoctonate ammonium salt, cytidine-5-monophospho-Nacetylneuraminic acid sodium salt, and darunavir were tested as inhibitors for the inhibition of binding between ACE2 and spike protein of the SARS-CoV2 using BPS bioscience ACE-2:SARS-CoV2 spike inhibitor screening ELISA assay kit.

**Figure A2:**
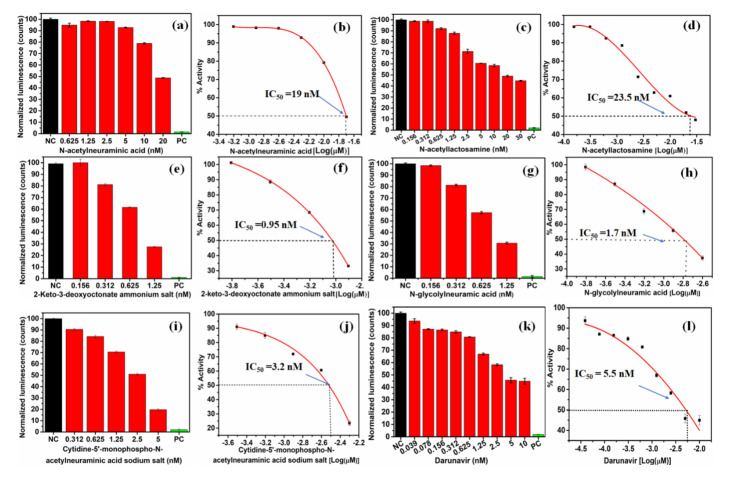
The ACE-2: SARS-CoV-2 Spike Inhibitor Screening assay was performed as described in the Materials and Methods section. The silicic acid family of n-acetyl-neuraminic acid, n-acetyllactosamine, 2-keto-3-deoxyoctonate ammonium salt, and cytidine-5-monophospho-N-acetylneuraminic acid were tested at various concentrations ranging from 0.1 to 30 nM to evaluate their ability to inhibit the binding of spike protein to immobilized ACE2 by using the ACE-2:SARS-CoV-2 Spike Inhibitor Screening Assay Kit. Chemiluminescence was measured using a FluoStar Omega Luminescence fluorescent microplate reader as per the ACE2:SARS-CoV-2 Spike Inhibitor Screening assay manufacture’s instructions. Normalized luminescence intensity (**a**,**c**,**e**,**g**,**i**,**k**) and ACE-2 and SARS-CoV-2 protein percentage inhibition activity (**b**,**d**,**f**,**h**,**j**,**l**) of (N-acetyl-neuraminic acid, N-glycolylneuraminic acid, 2-Keto-3-deoxyoctonate ammonium salt, n-glycolylneuramic acid, cytidine-5-monophospho-N-acetylneuraminic acid sodium salt, and darunavir. The experiment was performed twice for all compounds.

## Data Availability

The dataset used in this study can be downloaded from the BindingDB website, https://www.bindingdb.org (accessed on 16 October 2021). The code is available in the following repository, https://github.com/aidaty/UnbiasedDTI.

## Sample Availability

Samples of the compounds are available from the authors.

## Abbreviations

The following abbreviations are used in this manuscript:

DTI	Drug-target interaction
SMILES	Simplified Molecular-Input Line-Entry System
PSC	Protein Sequence Composition
ErG	Extended reduced graphs
ESPF	Explainable Substructure Partition Fingerprint

## Appendix A

In this study, predictions were tested using a standard laboratory binding assay to assess the validity of determined models. The assay consists of the inhibition of complex formation between the spike protein of SARS-CoV-2 and the ACE2 enzyme of the human cell. Adding an inhibitor compound prevents the binding and reduces the intensity of a luminescence signal. Seven inhibitor compounds including N-Acetyllactosamine, N-acetyl-neuraminic acid, 3 α ,6 α -Mannopentaose, N-glycolylneuraminic acid, 2-Keto-3-deoxyoctonate ammonium salt (from Fisher Scientific), cytidine-5-monophospho-N- acetylneuraminic acid sodium salt, and Darunavir (from Sigma Aldrich) were chosen to examine the sensitivity of the model predictions due to their similarity (or dissimilarity) to known inhibiting compounds introduced in the literature. ELISA-type ACE2: SARS-CoV-2 Spike Inhibitor Screening assay (Spike S1 RBD; BPS Bioscience Cat: 79936) was used according to the manufacturer’s instructions. Different concentrations ranging from 0.1 nM to 30 nM of the seven molecules were tested. The stock ACE2 protein was thawed on ice and diluted with phosphate-buffered saline (PBS) to 1 µg/mL. Then, 50 µL of diluted ACE2 was added to a nickel-coated 96-well plate and incubated at room temperature for 1 h on a low-speed shake-table. The plate was then rinsed three times with PBS and incubated in a blocking buffer for ten minutes. Subsequently, 10 µL of the candidate inhibitor compounds (dispersed to the required concentration in PBS) was added and incubated at room temperature for 1 h with slow shaking. As a positive control and blank measurement, an inhibitor buffer containing 5% DMSO was utilized. Spike protein was thawed in the same way as ACE2 and diluted in Assay Buffer 1 to 0.25 ng/mL. Spike protein was added to each well (except the blank wells) and shaken for an additional 1 h at room temperature on the shake table. Then, plates were rinsed three times with PSB and incubated for ten minutes in a blocking buffer. Anti-mouse Fc-HRP (horseradish peroxidase) was added, and the mixture was incubated for 1 h with steady slow shaking. Finally, after adding HRP substrate, chemiluminescence was measured using a FluoroStar Omega microplate reader. Compared to the negative control, a reduction in chemiluminescence is considered an interruption in the formation of the Spike-ACE2 complex, and as a result, the HRP chemiluminescence-producing substrate is washed away. To determine the concentration-dependent luminescence signals, spike protein standard curves were created by titration from 0.1 nM to 100 nM.
